# “Where does my menstruation come from?” Experiences and costs of menstrual hygiene management among Rwandese school girls

**DOI:** 10.1371/journal.pgph.0004152

**Published:** 2025-05-15

**Authors:** Kirsten Beata Dodroe, Lilian Nantume Wampande, Arlene Nishimwe, Mutesi Mukinisha, Janna M. Schurer

**Affiliations:** 1 Center for One Health, University of Global Health Equity, Butaro, Rwanda; 2 Division of Basic Medical Sciences, School of Medicine, University of Global Health Equity, Butaro, Rwanda; 3 Department of Infectious Disease and Global Health, Cummings School of Veterinary Medicine at Tufts University, North Grafton, Massachusetts, United States of America; Tata Institute of Social Sciences, INDIA

## Abstract

The Government of Rwanda recently instated a minimum menstrual hygiene management (MHM) package for all schools; however, coverage is not universal, and there remains little information on the availability or financial burden of MHM resources in rural and remote areas. This study sought to describe Rwandese secondary school girls’ perceptions and practices of MHM, their financial costs, and their access to MHM related infrastructure and products. A mixed-methods survey was conducted in three phases, namely (1) a quantitative questionnaire (2) a qualitative ‘Jar Voices’ activity, and (3) an observational Water, Sanitation and hygiene (WASH) checklist. One Lower Secondary School (LSS) of each type (private, public and government-aided) was randomly selected from each of Rwanda’s five provinces, and all LSS2 and LSS3 girls who reached menarche were invited to participate. Overall, 1,117 girls participated, including 351 (31.4%) from public schools, 543 (48.6%) from government aided schools and 223 (20.0%) from private schools. Only 35.7% of the girls correctly identified the origin of menstruation. The main source of information on menstruation was mothers. Most girls (82.5%) used single-use sanitary pads, and a small proportion (14.6%) relied on reusable cloth pads. The most identified barriers to accessing sanitary products were financial barriers. The average cost of menstrual products per cycle varied between RWF 3,100 in private schools, to RWF 4,000/4,300 in public/government-aided schools, and were covered mainly by parents. Most schools had gender-sensitive sanitation facilities, mainly pit latrines (86.7%), but only 53.3%, 33.33% and 20% provided water, soap and MHM commodities respectively. The results underscored the need for continuous menstrual education aimed at both girls and parents, and capitalizing on low-cost initiatives that can improve access to sanitary pads and WASH/sanitary facilities in schools.

## Introduction

Menstrual hygiene management (MHM) is defined as “using a clean menstrual management material to absorb or collect blood that can be changed in privacy as often as necessary for the duration of the menstruation period” [1]. Safe and dignified MHM is increasingly recognized as a health and human rights issue that is foundational to achieving gender equity [2–4]. Up to 75% of women and girls in low-income countries fail to manage their menstrual health comfortably each month, due to lack of access to safe, affordable and appropriate sanitary materials or private space to change these materials [5,6]. Managing menstruation at school can be particularly challenging if girls do not have access to basic MHM services. In a survey of 2,208 rural schools in Eastern and Southern Africa, fewer than 25% of schools offered clean water, adequate sanitation, or a private changing or washing facility for menstrual hygiene [7]. When menstruation is not managed safely, girls are at increased risk for urogenital infections, withdrawal from social activities and absenteeism from school leading to missed economic opportunities and oppression [8–12].

More than 50% of girls in sub-Saharan Africa miss school every month due to MHM-related issues, with absenteeism higher in rural areas [10,12,13]. In a recent Rwandese survey, 22.3% of rural high school girls reported menstrual related absenteeism [14]. Reasons for missing school included fear of leaking, lack of facilities for girls to change, and insufficient access to sanitary pads [14,15]. Moreover, commercial sanitary products were considered expensive, leading girls to use low-cost alternatives such as toilet paper or old cloth, which carry risks for hygiene and unwanted leaks. In some areas, financially deprived girls engage in transactional sex to earn money to care for their menstrual needs, increasing their risk to sexually transmitted infections (STI’s), and other threats [10,16].

In 2019, the Government of Rwanda committed to improving access and availability of sanitary pads by instating a minimum MHM package requirement for all schools [17]. This package included gender-separated toilets, a safe private room with sanitary pads, water, soap, spare underwear, pain medication and an attendant. However, many schools do not yet have these services, and there remains little information regarding the financial burden of MHM or the availability of sanitary products in rural and remote areas. Many cases of school dropouts for girls in Rwanda predominantly occur during secondary school. Therefore, this study aimed to describe the perceptions and practices of Rwandese Lower Secondary School (LSS) girls about MHM, to calculate the financial cost of MHM for girls, and to document the facilities and resources that shape girls’ experiences of menstruation. This data is vital to support Rwanda’s efforts to promote equitable educational and economic engagement for all students, and to enhance progress post the ‘MHM Ten Year Agenda’ [18].

## Methods

### Ethical approval

Ethical approval was gained from the University of Global Health Equity’s Institutional Review Board (protocol #: 141). Heads of Schools granted verbal, revocable permission for the research team to recruit participants from their respective schools. Informed written assent/consent was obtained from students and their parents/guardians.

### Study setting

Rwanda is a densely populated low-income country with a population of approximately 13 million people [19]. Rwandese households are grouped into four “Ubudehe” (i.e., socioeconomic) categories where Category 1 represents the poorest families and Category 4 represents the wealthiest. The poorest families receive government support, such as free universal health coverage. Out of pocket MHM costs include sanitary products, other products used during menstruation such as pain medication, and the transport to access these products. There are three types of schools: public, government aided, and private [20]. Public schools are funded entirely by the government and tend to educate lower income students. Government aided schools are funded by the government, the student’s family, and collective community associations, and serve primarily low to middle income students. At private schools, tuition is paid in full by the student’s family, and most students come from middle to high-income households. Schools are further categorized into day or boarding institutions, as well as single-sex or coeducational. LSS’s typically educate students aged 12–15 years old and may be combined with Senior Secondary Schools (SSS), which educate students aged 15–18 years. In 2018, there were 1,728 Secondary Schools in Rwanda, including 522 public, 892 government-aided, and 314 private schools. Public and government aided schools each enroll an average of 400 students, while private schools enroll approximately 250 students. Some schools provide MHM rooms, which are dedicated spaces for menstruating students to rest, clean their bodies, and change their sanitary products. These rooms may be equipped with MHM supplies, WASH facilities, or spare skirts for girls to change into if they bleed through their uniforms.

### Study design

This mixed methods, cross-sectional study included three activities: (1) a quantitative questionnaire administered to LSS students, followed by (2) a qualitative ‘Jar Voices’ activity, and finally (3) a descriptive WASH checklist completed by the researchers. Photographs of school WASH facilities were taken to provide context to the researchers’ observations.

### Study tools and data collection

To assess MHM practices, preferences, and financial burden, the team developed a multiple-choice questionnaire, written in English and translated to Kinyarwanda. The questionnaire was organized in five distinct parts (1) Basic demographic characteristics such as age, religion, school type, Ubudehe and parent/guardian occupation (2) Awareness and knowledge on menstruation (3) MHM products and Costs (4) Access to WASH/sanitary facilities (5) Absenteeism in the past 12 months. The Kinyarwanda questionnaire was pre-tested with four Rwandese female medical students to validate content, and then the questionnaire was back translated to English. Paper questionnaires were provided to participants in both English and Kinyarwanda.

To assess knowledge gaps, students were invited to write questions in Kinyarwanda or English about MHM or Sexual and Reproductive Health (SRH) on small pieces of paper, but without including their identifiable information. These questions were anonymously collected into a jar and answered by MHM educators on the study team. The purpose of the ‘jar voice’ exercise was to allow students to freely express their voices whether through a written text or diagram, thus allowing for a more personalized understanding of MHM issues during the time of discussion. To assess the presence of WASH facilities and materials, including water, toilets, and MHM rooms, one member of the study team completed an observational WASH checklist at each school.

The target population for the study was Rwandese girls enrolled in LSS2 or LSS3. This did not include younger class years, in which the girls may not have begun menstruating, or older class years, from which dropout rates are higher [21]. Using Cochran’s formula, the target sample size was 384 participants [22]. To obtain this sample size, one LSS of each type (private, public, government aided) was randomly selected in each of Rwanda’s five provinces to collect data from students of wide-ranging socioeconomic backgrounds. The Head of School of each selected school was contacted by telephone and email with information about the study. If they were unresponsive or did not want to participate, another school of the same type in the same province was randomly selected. All LSS2 and LSS3 girls at each participating school, regardless of menstruation status, were invited to participate in the quantitative study. Recruitment of participants took place from 25/03/2022 and ended on 11/07/2022.

### Data analysis

Following data collection, questionnaire responses were entered into a Google Form. Quality checks were conducted regularly by cross-checking anonymized photographs of randomly selected surveys and the entered results. Data from girls who self-identified as never menstruating were omitted during data cleaning. The data were analyzed using SPSS (IBM v. 28.0.1.1). Categorical data about MHM was reported descriptively with percentages and counts, disaggregated by school type. Differences among school types were evaluated using the Pearson’s Chi-Square Test when 80% of cell size >5 or the Fisher Exact Test when 20% of cell size <5. When variables allowed more than one response, each choice was analyzed independently as a binary ‘Yes/No’ response. Differences between school types were considered significant when a 2-sided p-value fell below 0.05.

Financial data was tested with a one-sample Kolmogorov-Smirnov test to test for normalcy. In the case of non-parametric data, costs were reported with medians and interquartile ranges, and in the local currency (2022 Rwandese Francs, RWF). Days of menstruation-related absenteeism were transformed into categorical variables, grouping 0 days (none), 1–4 days (few), 5–7 (moderate), and 8+ (many) days missed due to MHM. Photographs of WASH facilities and resources provided visual context to the physical setting of the schools.

Jar Voices questions were manually entered into an Excel spreadsheet and translated to English. To identify knowledge gaps, Jar Voice questions were analyzed and independently categorized by theme by LNW and KD. Final themes were developed after thorough discussion and agreement between the two researchers.

## Results

A total of 1,148 LSS2 and LSS3 girls from 15 schools agreed to participate. The analysis excluded the survey results of 31 girls who reported that they had not experienced menarche. Of the remaining 1,117 study participants, 351 (31.4%), attended public school, 543 (48.6%) attended a government aided school, and 223 (20.0%) attended private school (Table 1). The mean age of study participants was 16.3 years old (range: 13–21 years). Girls reported reaching menarche between the ages of 9 and 18 years (mean: 13.7 years). More girls in private schools belonged to the higher Ubudehe categories, compared to those in government aided or public schools.

**Table 1:** Demographics of female SS2 and SS3 study participants from 15 schools in Rwanda. (N = 1,117)^1^.

	Public (n = 351)	Government Aided (n = 543)	Private (n = 223)	Total	p-value
n (%)			
Religion^2^					
Christian	336 (95.7)	504 (92.6)	209 (93.7)	1049 (93.9)	0.049^*^
Muslim	8 (2.3)	24 (4.4)	11 (4.9)	43 (3.8)	
None	3 (0.9)	5 (0.1)	2 (0.1)	10 (1.0)	
No response	4 (1.1)	10 (1.8)	1 (0.4)	15 (1.3)	
Ubudehe Category					
1	24 (6.8)	52 (9.6)	9 (4.0)	85 (7.6)	0.001^*^
2	145 (41.3)	170 (31.3)	45 (20.2)	360 (32.2)	
3	170 (48.4)	295 (54.3)	143 (64.1)	608 (54.4)	
4	2 (0.6)	4 (0.4)	6 (2.7)	12 (1.0)	
No response	10 (2.8)	22 (4.1)	20 (9.0)	52 (4.7)	
Father/Male guardian’s occupation					
Unemployed	44 (12.5)	116 (21.4)	30 (13.5)	190 (17.0)	<0.001^*^
Homemaker	20 (5.7)	17 (3.1)	5 (2.2)	42 (3.8)	
Farmer	161 (45.9)	220 (40.5)	17 (7.6)	398 (35.6)	
Government	32 (9.1)	20 (3.7)	45 (20.2)	97 (8.7)	
Teacher	6 (1.4)	10 (1.8)	5 (2.2)	20 (1.8)	
Self-employed	58 (16.5)	89(16.4)	93 (41.7)	240 (21.5)	
Deceased	12 (3.4)	35 (6.4)	2 (0.9)	49 (4.4)	
No response	12 (3.7) 345	19 (3.5) 526	11 (4.9) 208	43 (3.8) 1079	
Mother/Female guardian’s occupation^2^					
Unemployed	45 (12.8)	128 (23.6)	41 (18.4)	214 (19.2)	0.001^*^
Homemaker	19 (5.4)	55 (10.1)	7 (3.1)	81 (7.3)	
Farmer	199 (56.7)	248 (45.7)	19 (8.5)	466 (41.7)	
Government	8 (2.3)	6 (1.1)	16 (7.2)	30 (2.7)	
Teacher	9 (2.6)	10 (1.8)	11 (4.9)	30 (2.7)	
Self-employed	49 (14.0)	69 (12.7)	104 (46.6)	222 (19.9)	
Deceased	5 (1.4)	7 (1.3)	3 (1.3)	15 (1.3)	
No response	5 (1.4) 339	16 (2.9) 539	7 (3.1) 208	28 (2.5)	

^1^“No response” not included in calculations

^2^Fisher’s Exact test conducted due to small cell counts

*Statistically significant p-value

### Perceptions of menstruation

Across all schools, girls often first learned about menstruation from their mothers or female guardians (55.9%; Table 2), and usually (79.1%) prior to menarche (p-value = 0.159). As they got older, girls continued to seek MHM information from their mother or female guardian (60.0%), friends (33.6%), or teacher (19.1%). Most girls identified menstruation as a normal process (93.6%, p-value = 0.665); however, only one-third could correctly identify the source of menstrual blood as the uterus (35.7%).

**Table 2:** Biological knowledge and social perceptions of schoolgirls in Rwanda about menstrual hygiene and sexual reproductive health (N = 1,117)^1^.

	Public (n = 351)	GovernmentAided (n = 543)	Private (n = 223)	Total	p-value
	n (%)				
Taught about menstruation before menarche					
Yes	270 (76.9)	425 (78.3)	188 (84.3)	883 (79.1)	0.159
No	72 (20.5)	106 (19.5)	34 (15.2)	212 (19.0)	
No response	9 (2.6)	12 (2.2)	1 (0.4)	22 (2.0)	
First source of information about menstruation^3^					
Health care provider	21 (6.0)	39 (7.2)	5 (2.2)	65 (5.8)	0.029^*^
Mother/female guardian	210 (59.8)	292 (53.8)	122 (54.5)	624 (55.9)	0.190
Father/male guardian	30 (8.5)	26 (4.8)	10 (4.5)	66 (5.9)	0.040^*^
Teacher	159 (45.3)	196 (36.1)	87 (38.8)	442 (39.6)	0.023^*^
Friend	96 (27.4)	131 (24.1)	28 (12.5)	255 (22.8)	<0.001^*^
Books	33 (9.4)	68 (12.5)	18 (8.0)	119 (10.7)	0.127
Media (TV, social media)	23 (6.6)	118 (21.7)	16 (7.1)	157 (14.1)	<0.001^*^
No response	3 (0.9)	16 (2.9) 886	6 (2.7)	25 (2.2)	
Most frequently used source of information about menstruation^3^					
Health care provider	41 (11.7)	76 (14.0)	21 (9.4)	138 (12.4)	0.194
Mother/female guardian	204 (58.1)	324 (59.6)	142 (63.4)	670 (60.0)	0.407
Father/male guardian	24 (6.8)	26 (4.8)	10 (4.5)	60 (5.4)	0.334
Teacher	68 (19.4)	112 (20.6)	33 (14.7)	213 (19.1)	0.173
Friend	131 (37.3)	182 (33.5)	62 (27.7)	375 (33.6)	0.063
Books	42 (12.0)	56 (10.3)	11 (4.9)	109 (9.8)	0.018^*^
Media (TV, social media)	36 (10.3)	91 (16.7)	16 (7.1)	143 (12.8)	<0.001^*^
No response	8 (2.3)	18 (3.3)	6 (2.7)	32 (2.9)	
Thinks menstruation is a normal process^4^					
Yes	329 (93.7)	509 (93.7)	208 (93.3)	1046 (93.6)	0.665
No	6 (1.7)	13 (2.4)	6 (2.7)	25 (2.2)	
Unsure^4^	15 (4.3)	17 (3.1)	6 (2.7)	38 (3.4)	
No response	1 (0.3)	4 (0.7)	3 (1.3)	8 (0.7)	
Origin of menstrual blood^2^^,^^4^					
Abdomen	21 (6.0)	10 (1.8)	2 (0.9)	33 (3)	0.002^*^
Bladder	7 (2.0)	10 (1.8)	6 (2.7)	23 (2.1)	
Vagina	208 (59.3)	280 (51.6)	120 (53.8)	608 (54.4)	
Uterus/Womb	105 (29.9)	209 (38.5)	85 (38.1)	399 (35.7)	
I don’t know	3 (0.9)	6 (1.1)	2 (0.9)	12 (1.1)	
No response	7 (2.0)	28 (5.2)	7 (3.1)	42 (3.8)	

^1^“No response” not included in Pearson’s Chi Square and Fisher’s Exact tests

^2^Fisher’s Exact test conducted due to small cell count

^3^Respondents could select multiple answers

^4^“Unsure” and “I don’t know” included in Pearson’s Chi Square and Fisher’s Exact tests

*Statistically significant p-value

### Menstrual hygiene practices and preferences

Girls most frequently used single-use sanitary pads to absorb their menstrual fluid (89.3%; Table 3). The use of products did not vary significantly among school types, except for the use of reusable pads, which were used most frequently by private school girls (20.1%). The primary reasons for choosing these products included cost (25.0%), safety (20.1%), and comfort (19.2%). Girls from private schools were 50% more likely to select products based on their comfort (24.26%) than girls from government aided schools (16.4%). At all school types, most girls preferred using single use pads (82.5%).

**Table 3:** Menstrual hygiene practices and preferences of schoolgirls in Rwanda (N = 1,117)^1^^.^

	Public (n = 351)	Government Aided (n = 543)	Private (n = 223)	Total	p-value
	n (%)				
Most frequently used product during menstruation^3^					
Sanitary Pads	314 (89.5)	493 (90.8)	191 (85.3)	998 (89.3)	0.111
Tampons^2^	5 (1.4)	11 (2.0)	6 (2.7)	22 (2.0)	0.563
Reusable cloth or pad	38 (10.8)	80 (14.7)	45 (20.1)	163 (14.6)	0.008*
Underwear	55 (15.7)	104 (19.2)	43 (19.2)	202 (18.1)	0.365
Toilet paper	28 (8.0)	34 (6.3)	15 (6.7)	77 (6.9)	0.610
Natural materials	0 (0)	7 (1.3)	1 (0.4)	8 (0.7)	0.132
No response	8 (2.3)	3 (0.6)	1 (0.4)	12 (1.1)	
Preferred product during menstruation^2^					
Sanitary Pads	285 (81.2)	453 (83.4)	184 (82.5)	922 (82.5)	0.170
Tampons	42 (12.0)	40 (7.4)	19 (8.5)	101 (9.0)	
Reusable cloth or pad	7 (2.0)	9 (1.7)	3 (1.3)	19 (1.7)	
Underwear	5 (1.4)	21 (3.9)	7 (3.1)	33 (3.0)	
Toilet paper	3 (0.9)	8 (1.5)	5 (2.2)	16 (1.4)	
None	3 (0.9)	3 (0.6)	3 (1.3)	9 (0.8)	
No response	6 (1.7)	3 (0.6)	1 (0.4)	16 (1.4)	
Reasons for choosing most frequently used product ^3^					
Comfort	70 (19.9)	89 (16.4)	55 (24.6)	214 (19.2)	0.027*
Safety	68 (19.3)	119 (21.9)	37 (16.5)	224 (20.1)	0.230
Cost	82 (23.3)	145 (26.7)	52 (23.2)	279 (25.0)	0.432
Availability	45 (12.8)	77 (14.2)	33 (14.7)	155 (13.9)	0.768
Ease of re-use	24 (6.8)	42 (7.7)	16 (7.1)	82 (7.3)	0.001*
Ease of disposal	26 (7.4)	48 (8.8)	28 (12.5)	102 (9.1)	0.107
Mother’s preference	32 (9.1)	58 (10.7)	22 (9.8)	112 (10.0)	0.746
Satisfaction with most used product					
Very satisfied	178 (50.7)	248 (45.7)	96 (43.0)	522 (46.7)	0.010*
Somewhat satisfied	112 (31.9)	168 (30.9)	87 (39.0)	367 (32.9)	
Not satisfied	29 (8.3)	93 (17.1)	29 (13.0)	151 (13.5)	
Prefer not to answer	16 (4.6)	19 (3.5)	4 (1.8)	39 (3.5)	
No response	15 (4.3)	14 (2.6)	7 (3.1)	36 (3.2)	
How often can you access the sanitary product of your preference?					
Always	189 (51.3)	167 (30.8)	162 (72.6)	509 (45.6)	<0.001*
Sometimes	149 (42.5)	330 (60.8)	57 (25.6)	536 (48)	
Never	13 (3.7)	39 (7.2)	2 (0.9)	54 (4.8)	
No response	9 (2.6)	5 (0.9)	2 (0.9)	16 (1.4)	
Where sanitary products are obtained while at school^3^					
Shop	167 (47.6)	214 (39.3)	135 (60.3)	516 (46.2)	<0.001*
Family member	50 (14.2)	66 (12.1)	41 (18.3)	157 (14.1)	<0.001*
Teacher/School	125 (35.6)	282 (51.8)	27 (12.1)	434 (38.9)	<0.001*
Other	10 (2.8)	28 (5.1)	11 (4.9)	49 (4.4)	0.234
No response	13 (3.7)	19 (3.5)	10 (4.5)	42 (3.8)	
Where sanitary products are obtained while at home^3^					
Shop	295 (84.0)	388 (71.5)	174 (77.7)	857 (76.7)	<0.001*
Family member	57 (16.2)	132 (24.3)	59 (26.3)	248 (22.2)	0.004*
Teacher/School^2^	7 (2.0)	15 (2.8)	2 (0.9)	24 (2.1)	0.263
Other	10 (2.8)	19 (3.5)	0 (0)	29 (2.6)	0.112
No response	19 (5.4)	31 (5.7)	9 (4.3)	59 (5.3)	
Source of money for sanitary products^3^					
Parents/guardians	302 (86.0)	453 (83.3)	214 (95.5)	969 (86.8)	<0.001*
Friends	31 (8.8)	60 (11.0)	7 (0.3)	98 (8.8)	0.002*
Other	9 (2.6)	49 (9.0)	2 (0.1)	60 (5.3)	0.003*
No response	23 (6.6)	22 (4.0)	6 (0.3)	51 (4.6)	
Barriers that prevented girls from buying sanitary products^3^					
No money	201 (57.3)	418 (76.8)	66 (29.5)	685 (61.3)	<0.001*
Embarrassed to buy	48 (13.7)	45 (8.3)	50 (22.3)	143 (12.8)	<0.001*
Unavailable at the shop	40 (11.4)	23 (4.2)	25 (11.2)	88 (7.9)	<0.001*
No barriers^4^	33 (9.4)	24 (4.4)	52 (23.2)	109 (9.8)	0.001*
No response	51 (14.5)	45 (8.3)	38 (17.0)	134 (12.0)	

^1^“No response” not included in Pearson’s Chi Square and Fisher’s Exact tests

^2^Fisher’s Exact test conducted due to small cell count

^3^Respondents could select multiple answers

^4^“No barriers” included in Pearson’s Chi Square test

* Statistically significant p-value

Students at government aided schools had the greatest trouble accessing their preferred product and were least satisfied with their sanitary products compared to girls at public and private schools. Girls obtained money for these products from their parents (86.8%), friends (8.8%), and other sources (5.3%) such as teachers, personal income, or savings. Lack of money was the greatest barrier to buying sanitary products.

### Financial cost of MHM

The total median amount spent per menstrual cycle on MHM-related products varied significantly among schools (p-value = 0.036), with girls paying a median of 4,000 RWF (IQR: 1,600–11,000 RWF) at public schools, 4,300 RWF (IQR: 2,000–10,000 RWF) at government aided schools, and 3,100 (IQR: 1,000–8,550 RWF) at private schools (Table 4). Girls at private schools spent a median of 0 RWF on transportation to acquire sanitary products, contributing to their lower median cost of menstruation per cycle. Girls at public and government aided schools consistently spent more on additional products purchased during menstruation than girls at private schools (p-values < 0.008).

**Table 4:** Median costs (RWF) of menstrual hygiene products and transportation to obtain sanitary products for schoolgirls in Rwanda in 2022^1^^,^^5^.

	Public	Government Aided	Private	Total	p-value
	Median (IQR)				
Travel cost to obtain sanitary products					
From school	700 (0-1000)	900 (0-1000)	0 (0-1000)	700 (0-1000)	0.008^*^
From home	800 (0-1000)	1000 (0-1000)	100 (0-1000)	800 (0-1000)	0.038^*^
Amount paid for sanitary products per menstrual cycle					
Typical	1200 (800-3000)	1625 (1000-5000)	2000 (925-3875)	1800 (1000-4000)	0.427
Amount paid for additional products per menstrual cycle^2^					
Typical	1000 (0-3500)	1000 (500-4000)	800 (0-2500)	1000 (0-3500)	0.001^*^
Median cost of menstruation per cycle^3^					
	4000 (1600-11,000)	4300 (2000-10,000)	3100 (1000- 8550)	4000 (1625-10,000)	0.036^*^
Annual cost of menstruation^4^					
	48,000	51,600	37,200	48,000	

^1^“No response” not included in calculations

^2^Additional products included pain medication or herbal medications

^3^Variable is a summation of travel cost to obtain products from home, travel costs to obtain products from school, typical amount paid for sanitary products, and typical amount paid for additional products per menstrual cycle

^4^Variable is the median cost of menstruation per cycle multiplied by 12

^5^Sample sizes vary for each question due to no response

*Statistically significant p-value

### WASH facilities and resources

**Fig 1:**
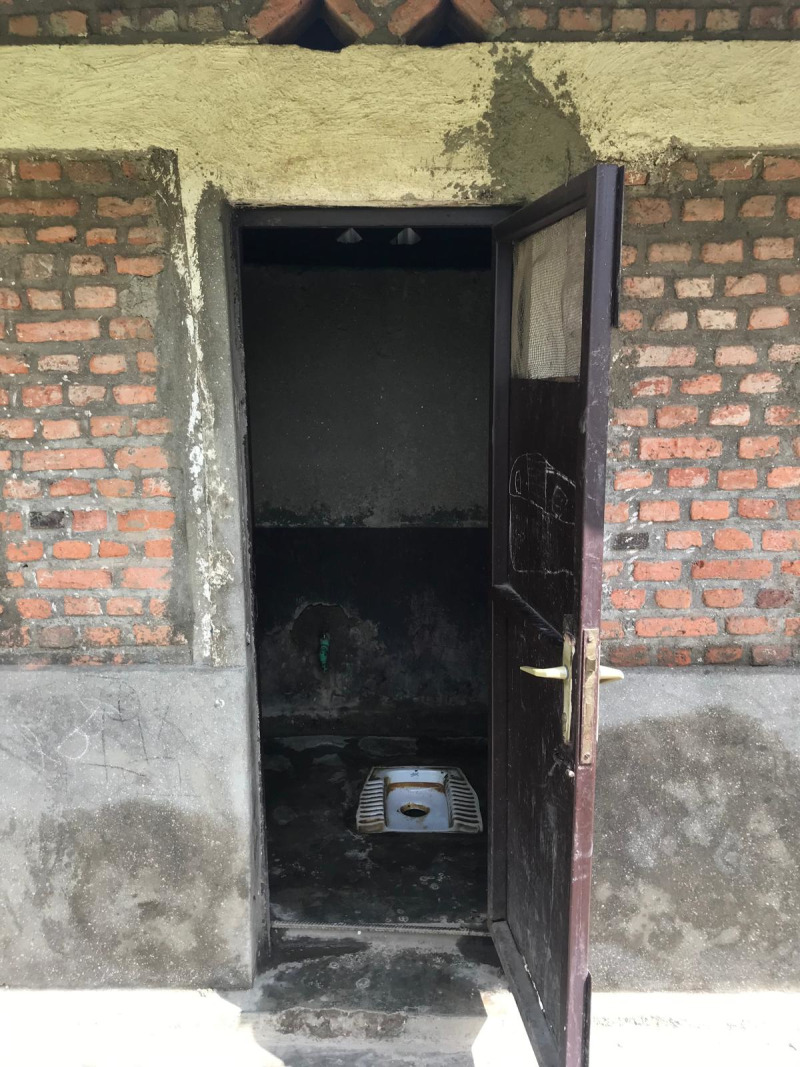
Pit latrine at a government aided school.

**Fig 2:**
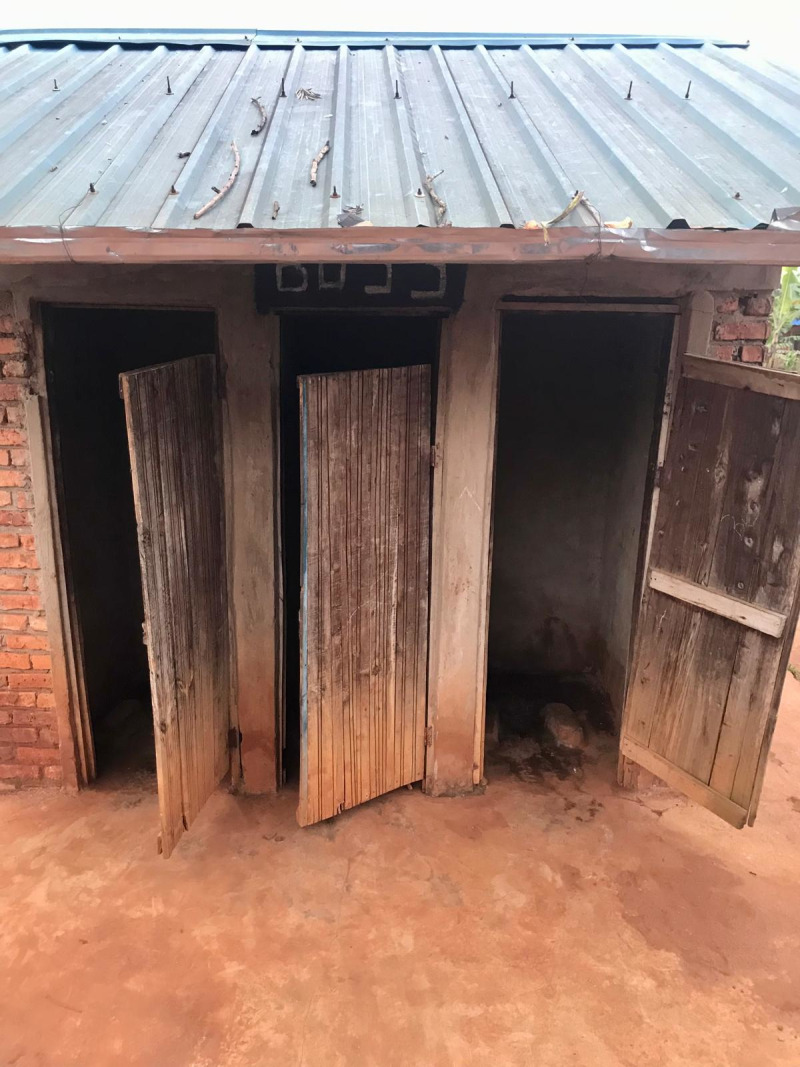
Pit latrine at a public school.

**Fig 3:**
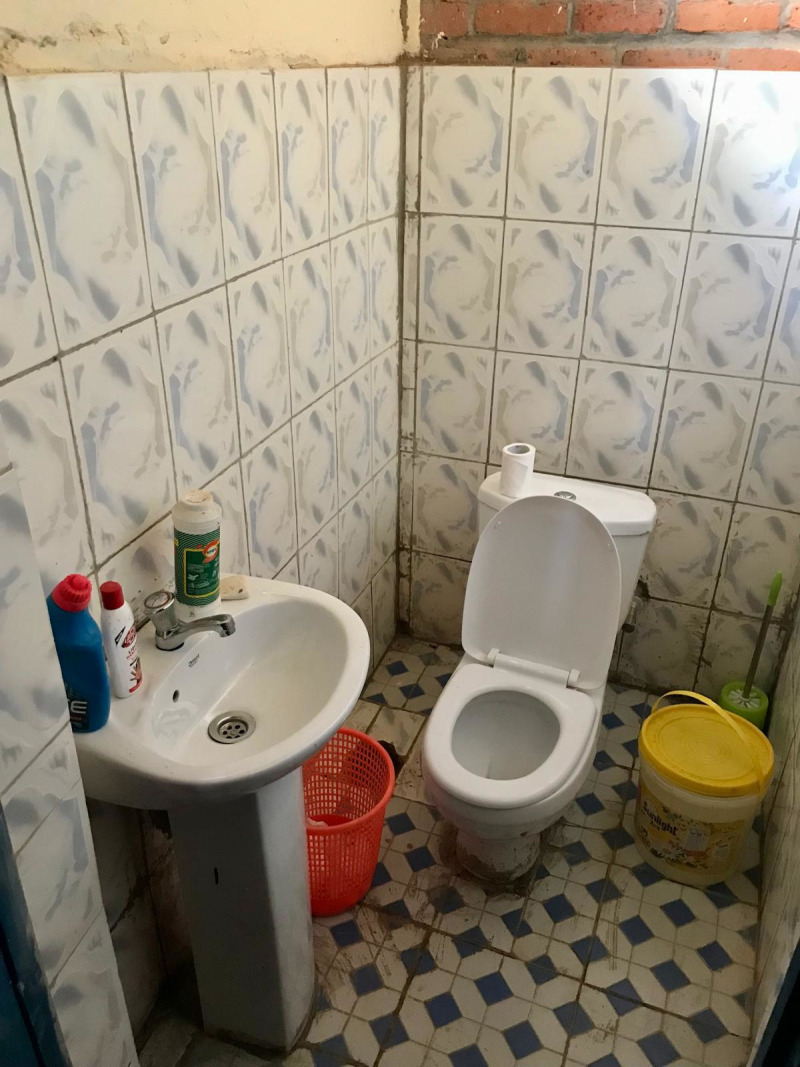
Flush toilet in an MHM room at a government aided school.

**Fig 4:**
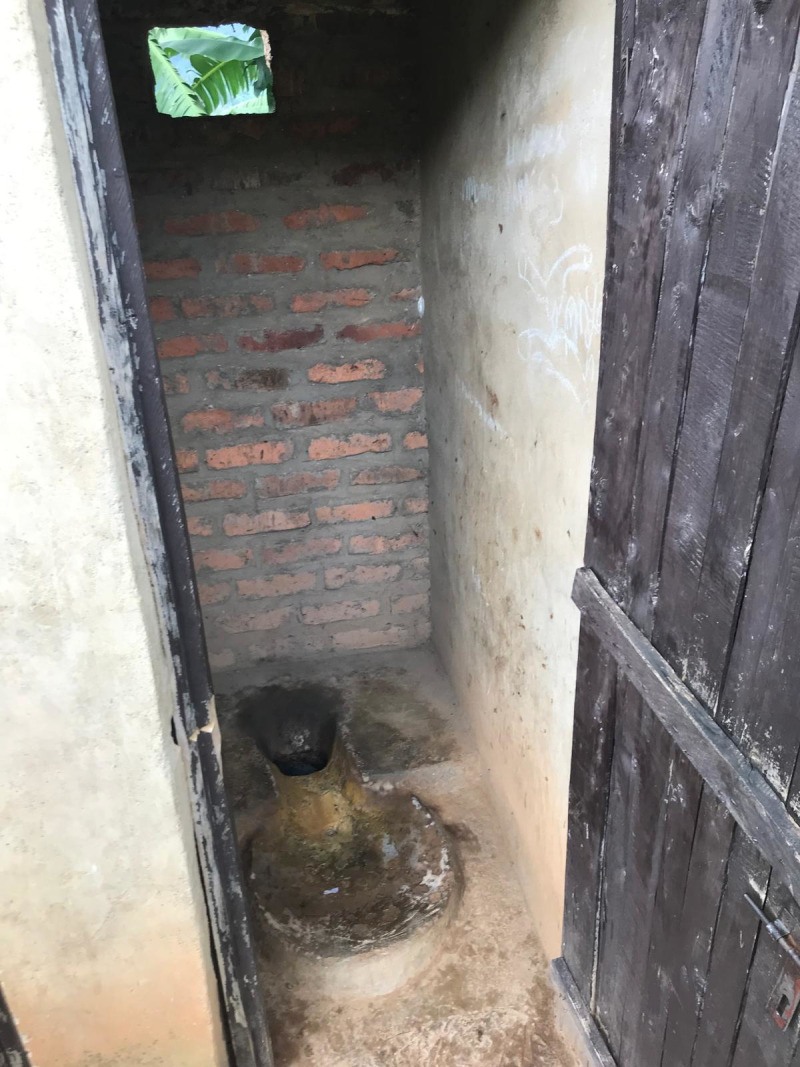
Pit latrine at a private school.

All schools had sanitation facilities separated by gender, most of which were pit latrines (n = 13, 86.7%) as shown in the figures below (Figs 1–4).

Approximately half of the schools (53.3%) had a water source present at the time of the site visit. Of these, water was sourced from a faucet (n = 5), a bucket (n = 2), and a tippy tap (n = 1). One-third of schools (33.3%) had soap available in latrine areas. Two of the 15 schools provided toilet paper, while at the remaining 13 schools, girls were expected to supply their own.

**Fig 5:**
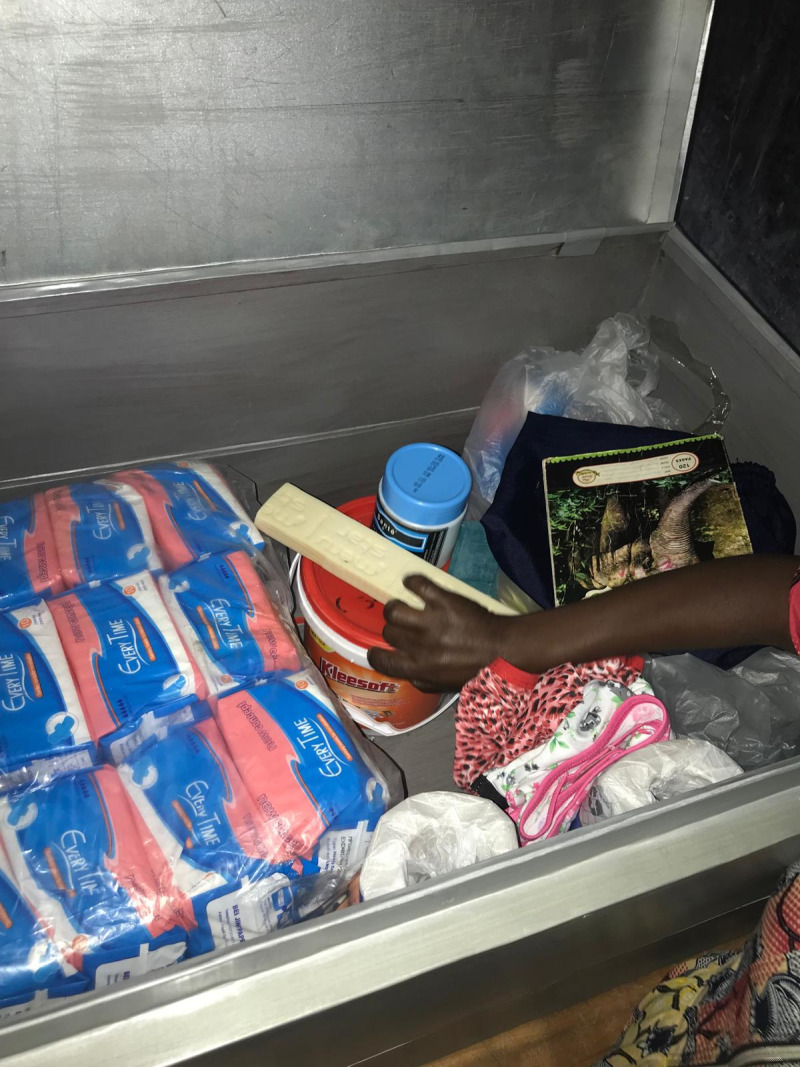
Box with sanitary pads, body and clothing soap, clean underwear, toilet paper, and logbook in an MHM room at a government aided school.

**Fig 6:**
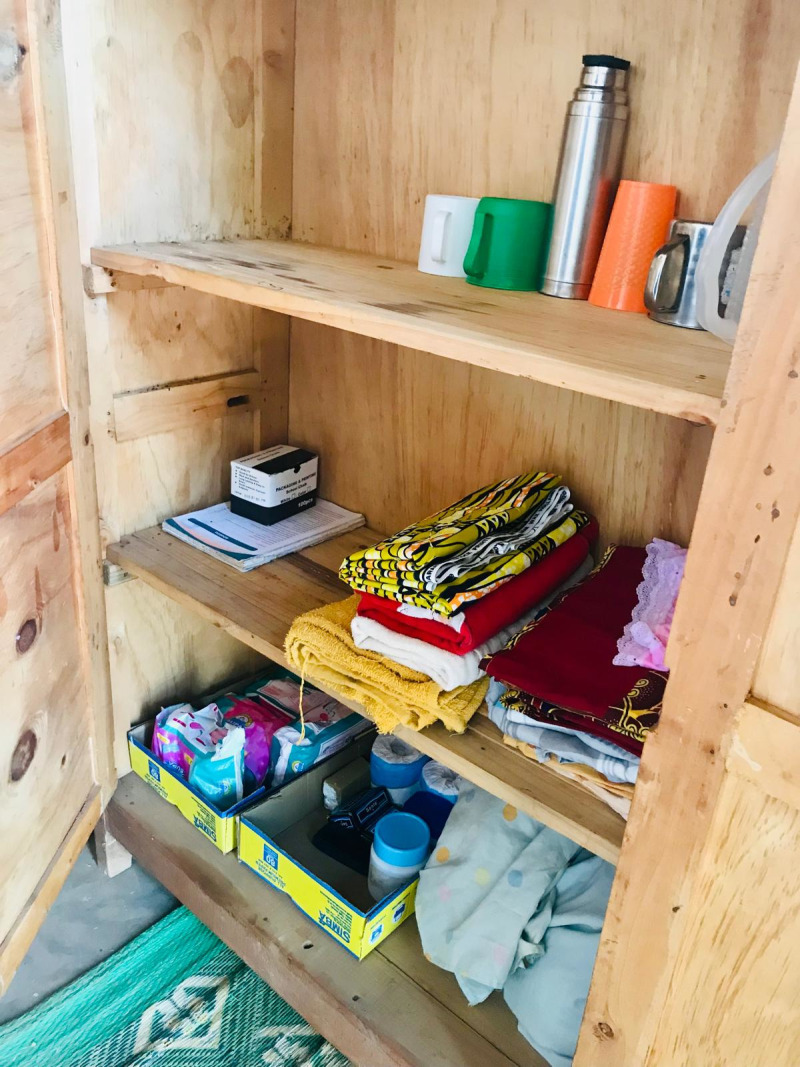
Cabinet with sanitary pads, clean underwear, clean skirts, soap, pain medications, towels, and cups for water at a private school in an MHM room.

**Fig 7:**
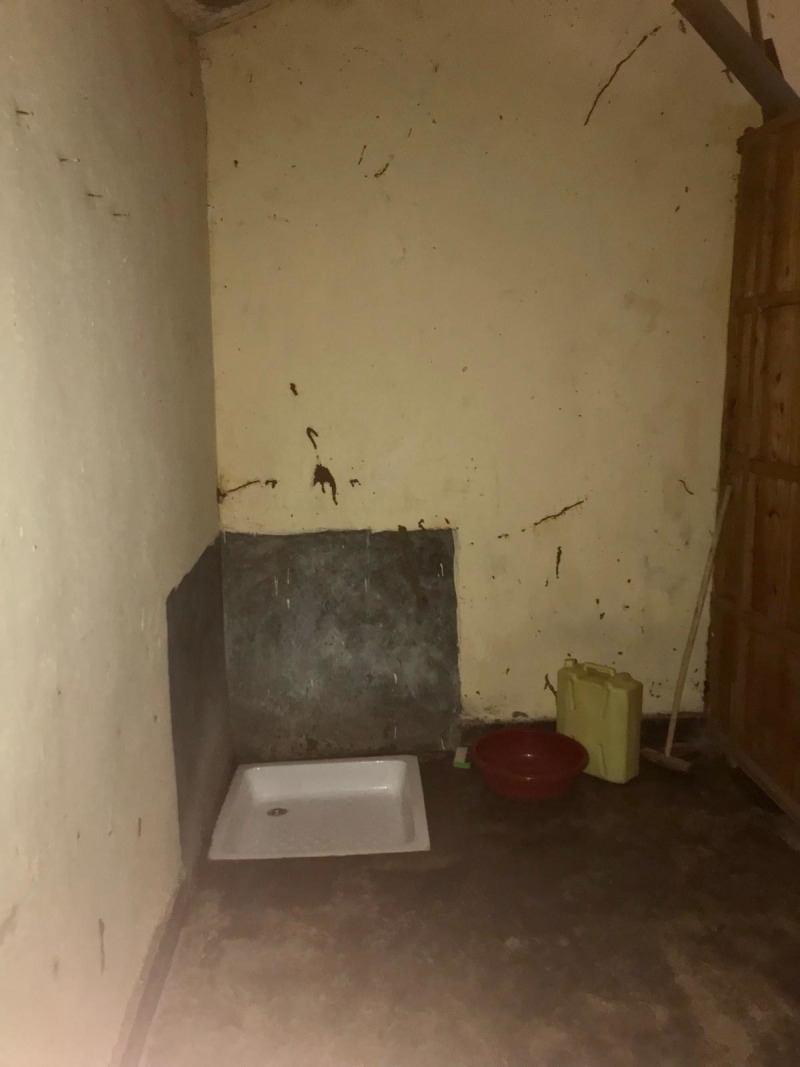
Water, sanitation and hygiene facility in a public school.

**Fig 8:**
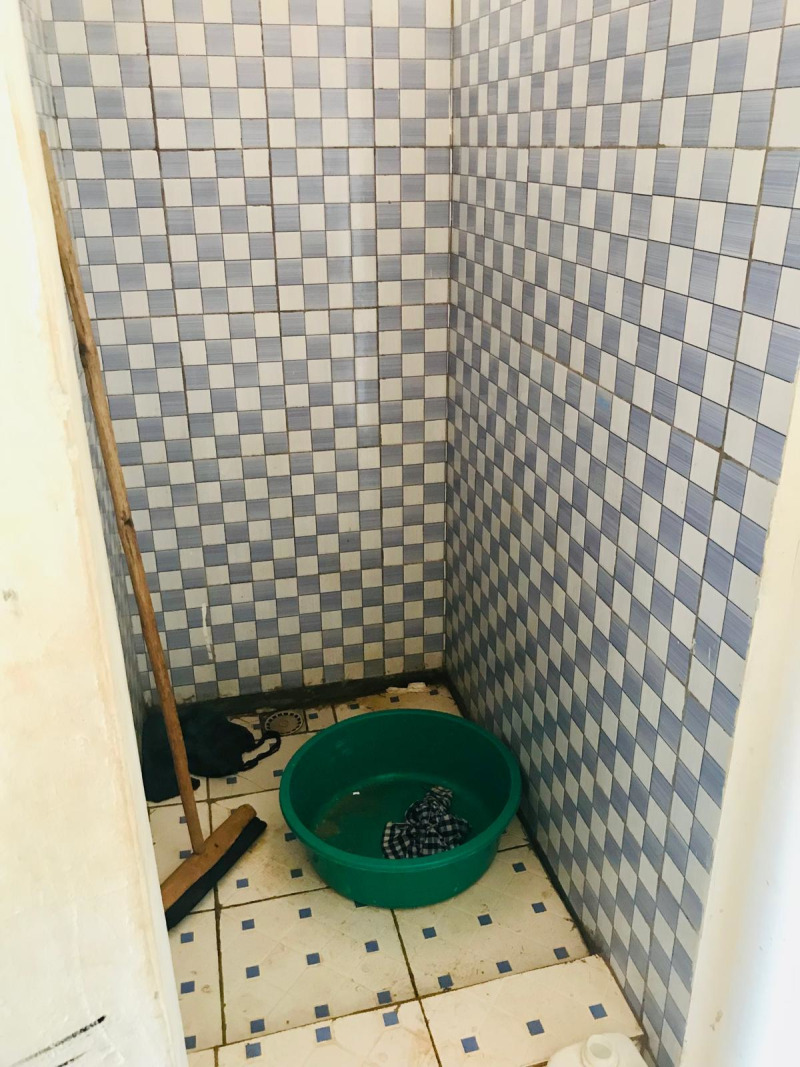
Water, sanitation and hygiene facility in a government aided school.

Ten of the 15 schools had MHM rooms. These rooms varied in their provision of MHM supplies and WASH infrastructure as shown in the figures below (Figs 5–8).

Schools with MHM rooms in most cases provided a private space for girls to manage their menstrual hygiene. At three locations, schools provided free disposable sanitary pads, toilet paper, underwear, soap, and pain medications to the girls. Girls primarily disposed of their sanitary products in the latrine (78.2%). In addition to throwing used sanitary products in the latrine, girls also threw them in the trash (9.6%) or disposed of them in other ways (6.5%) such as burning or throwing them into a receptacle inside a menstrual hygiene room (Table 5).

**Table 5:** WASH practices and access to resources for Rwandese school girls during menstruation (N = 1,117) ^1^.

	Public (n = 351)	Government Aided (n = 543)	Private (n = 223)	Total (N = 1,117)	p-value
	Count (%)				
At school, do you always have access to these items?^2^					
Clean sanitary product	242 (68.9)	343 (63.1)	162 (72.3)	747 (66.9)	0.025^*^
Soap	190 (54.1)	277 (50.9)	136 (60.7)	603 (54.0)	0.042^*^
Clean water	187 (53.3)	308 (56.6)	140 (62.5)	635 (56.8)	0.081
Private changing area	219 (62.4)	357 (65.6)	139 (62.1)	715 (64.0)	0.501
No response	22 (6.3)	52 (9.6)	15 (6.7)	89 (8.0)	
At home, do you always have access to these items?^2^					
Clean sanitary product	228 (64.8)	341 (62.7)	173 (77.2)	742 (66.4)	<0.001^*^
Soap	211 (59.9)	354 (65.1)	149 (66.5)	714 (63.9)	0.318
Clean water	224 (63.6)	391 (71.9)	159 (71.0)	774 (69.3)	0.027^*^
Private changing area	225 (63.9)	368 (67.6)	153 (68.3)	746 (66.8)	0.425
No response	17 (4.8)	33 (6.1)	13 (5.8)	63 (5.6)	
At school, where do you dispose of used sanitary products?					
Latrine	300 (85.5)	482 (88.8)	92 (41.3)	874 (78.2)	<0.001^*^
Trash	27 (7.7)	23 (4.2)	57 (25.6)	107 (9.6)	
Other	9 (2.6)	14 (2.6)	64 (28.7)	87 (7.8)	
No response	15 (4.3)	20 (3.7)	10 (4.5)	45 (4)	
At home, where do you dispose of used sanitary products?					
Latrine	313 (89.2)	508 (93.6)	151 (67.7)	972 (87.0)	<0.001^*^
Trash	21 (6.0)	13 (2.4)	40 (17.9)	74 (6.6)	
Other	2 (0.6)	9 (1.7)	18 (8.0)	29 (2.7)	
No response	15 (4.3)	13 (2.4)	13 (5.8)	41 (3.7)	
Frequency of changing sanitary product during menstruation					
Less than once a day	15 (4.3)	18 (3.3)	2 (0.9)	35 (3.1)	0.001^*^
Once a day	57 (16.2)	106 (19.5)	18 (8.1)	181 (16.2)	
2-3 times a day	229 (65.2)	330 (60.8)	155 (69.5)	714 (63.9)	
4+ times a day	32 (9.1)	68 (12.5)	35 (15.7)	135 (12.1)	
Prefer not to say	2 (0.6)	6 (1.1)	4 (1.8)	12 (1.1)	
No response	14 (4.0)	15 (2.8)	6 (2.7)	35 (3.1)	

^1^“No response” not included in Pearson’s Chi Square and Fisher’s Exact tests

^2^Respondents could select multiple answers

*Statistically significant p-value

### MHM knowledge gaps

Study participants submitted 1,913 questions during the Jar Voices activity. Analysis of the questions yielded five themes to describe the participant’s MHM and sexual reproductive health (SRH) experiences and curiosities, including: (1) MHM, cycles, and tracking (37% of questions); (2) SRH education (23%); (3) MHM pain and pain management (23%); (4) sanitary products and uses (8%); (5) and labia pulling (3%).

#### Theme 1 - MHM, cycles, and tracking.

This theme related to physiological and biological processes of menstruation, hygiene and self-care during menstruation, and associated symptoms of menstruation, including bloating and vaginal discharge. Students asked questions about irregularities in their menstrual cycle and how they could best track their periods. “Where does my period come from?”; “Is it bad if I don’t menstruate for three months even though I have gotten my period?”.

#### Theme 2 - Sexual and reproductive health education.

These questions centered on biological and social factors pertaining to infertility, pregnancy, sexual activity, virginity, and boys. Questions such as “can a woman get pregnant during her period”, or “do boys know if I am on my period” appeared frequently.

#### Theme 3 - Pain and pain management.

Commonly, girls asked about the safety and efficacy of pain medications. Participants asked questions about the cause of menstrual pain and effective strategies for pain management. For example, girls asked, “can painkillers help me not have pain?” and “will I get addicted to pain medications?”.

#### Theme 4 - Sanitary products and uses.

Girls frequently asked questions about what certain sanitary products are: “what are tampons?”, and how to use them: “how do I use a sanitary pad”? Girls asked where they could access products and what they could do if they could not afford them.

#### Theme 5 - Labia pulling.

This theme is related to traditional Rwandese sexual practices of “*guca imyeyo*”, or labia pulling, during which a female relative pulls to elongate a pubescent girl’s labia. Traditionally, this practice is seen to increase sexual pleasure for her and her future sexual partner. Girls asked what the practice is, what its effects are, and if they could still get married without participating in the practice: “can a girl have a husband if she did not participate in *guca imyeyo*?”

## Discussion

Our estimate of annual MHM costs (48,000 2022RWF; $47 2022USD per girl), amounted to 5% of an average annual Rwandese income ($940 2022USD) [23]. Similar estimates were reported in Uganda, where sanitary products for one girl were found to cost up to 10% of household income ($930 2022USD) [7,23]. In this study, girls from public/government aided schools incurred consistently higher costs on pads and additional products like soap, medications, and transport. Previous surveys conducted among girls from low-income backgrounds in Africa reported similar financial challenges [6,24–26]. However, these studies did not provide insights about who often paid for the MHM associated costs. Our analysis indicated that most of the costs associated with MHM were borne by parents, most of whom were farmers, although friends and teachers also provided some financial support. Previous studies done in low-income contexts indicated positive association between parents’ socio-economic status and menstrual related school dropout rates, poor hygienic practices and risky sexual behaviors among girls [16,25]. This suggests that girls attending public or government aided schools require the most support in achieving universal access to MHM.

In this study, girls mostly used and preferred disposable sanitary pads, and few (14.6%) relied on reusable cloth pads. However, the majority expressed that disposable pads were very expensive and hard to obtain. This aligned with previous findings from Kenya, where 71% of schoolgirls who preferred to buy disposable pads were hindered by costs [27], and Rwanda, where sanitary products were highly priced and out of reach for most rural low-income girls [13]. Given that this study was conducted in rural settings, this finding raises concerns about how girls manage to acquire disposable pads. A previous study reported girls engaging in risky sexual behaviors to obtain money for MHM products, with resulting consequences on their health and wellbeing [10]. It is therefore urgent to educate both girls and parents on other safe and affordable alternative products that can be used to manage menstruation, while also improving accessibility to these alternatives. Past studies have indicated that reusable pads can be safe and affordable. In three surveys, schoolgirls perceived reusable pads as less costly, more reliable, less difficult to change and less disgusting while cleaning [28–30]. The Government of Rwanda could therefore consider providing schools with reusable cloth pads or hosting workshops to teach girls to create reusable pads out of clean fabric collected from local tailors. These workshops could reduce cloth waste, provide girls with low-cost pads, and create an opportunity for workshop facilitators to share information about MHM. Sustainable Health Enterprises (SHE) and United Nations Population Fund (UNFPA) have already initiated the washable/reusable pads initiatives among some low-income households in Rwanda and their existing work and experience can inform better ways of delivering reusable pads [31]. However, caution must be taken when promoting re-usable pads as they can be a source of urogenital infections if not washed with clean water, changed at least three times per day, and dried properly [13]. Since only 56% of girls had access to clean water at school, programs to distribute reusable pads must be paired with initiatives to improve schools water infrastructure. Additionally, to reduce social stigma associated with reusable pads, girls must have private places to wash and dry pads [28–30].

Our team observed some effort to provide basic MHM facilities as mandated by the Government of Rwanda policy at all fifteen schools. Ten of the fifteen schools provided additional MHM rooms, with some (20%) providing basic commodities like sanitary pads, underwear, soap, toilet paper and pain medications to girls. Sanitation facilities (mostly pit latrines), separate for boys and girls, were present in 100% of the schools. According to past studies, access to, and availability of gender-sensitive menstrual hygiene facilities promoted comfort and confidence for girls to attend school on days when they are menstruating [32–34]. However, having separate toilets for boys and girls alone is not enough without adequate WASH commodities such as soap, clean water and proper waste disposal facilities [33,35]. In this study, about 53.3% of the schools we surveyed had a water source such as faucets, buckets or tippy taps. In 56.8% of our responses, girls said that they had access to clean water in school. Soap was found in only one third of the schools. A previous Rwandese survey also found that a high percentage (95.4%) of girls bathed during menstruation, but fewer (58.8%) washed with water and soap [14]. Studies from other low-income contexts also underscored WASH challenges during menstrual hygiene management [10,12,16]. For the Rwandese context, inadequate water and soap could explain why very few girls (12.1%) changed their sanitary pads as often as required. More attention is needed to translate government policies regarding the improvement of WASH at schools to ensure girls can wash their bodies and reusable pads during menstruation. Compliance with these policies could be monitored through UNICEF’s Joint Monitoring Program of MHM and WASH in schools [36].

In recent years, there has been increased interest in the link between MHM and environmental health. Our respondents disposed of their wasted sanitary products in pit latrines, and where absent, girls burned their pads. This concurs with findings from other sub-Saharan African contexts, including Zambia, where girls disposed of used pads in pit latrines due to fear of witchcraft [12]. Sanitary wastes contribute to several ecological issues, which include landfill overflow, pollution, and safety hazards. In addition, sanitary pads contain plastic compounds and bleached cotton that take decades to degrade naturally, so when thrown into open pits like latrines, they may disrupt the natural decomposition of organic waste [37,38]. Also, disposing of pads by burning can introduce toxic gases into the air through the release of furans, dioxins, mercury, and polychlorinate [39]. Teaching schoolgirls to make their own reusable pads would reduce the amount of waste generated by MHM and minimize the pollution caused by menstrual waste.

Girls in this study received menstrual information from teachers (19.1%) or healthcare providers (12.4%) compared to mothers (60%) or friends (33.6%). This result aligns with previous studies in similar settings [10,12,14,40], except for one study in Ethiopia where teachers were the main source of information [41]. Given that varied perceptions about menstruation exist [42], this raises concerns of whether girls get correct and timely information on menstrual hygiene practices. Our analysis indicated that more than half of the girls did not know the origin of menstruation. Girls were also skeptical on issues like tracking their menstrual cycle, getting pregnant, infertility, pain-regulation, and types of pads. To some extent, this indicated ill-preparedness and lack of adequate guidance on MHM. When girls do not have adequate understanding of the menstrual process, they may find it difficult to embrace it as a normal and natural process, thus may develop feelings of shame and embarrassment. Therefore, consistent and reliable information on MHM is crucial for every girl to dissipate any misconceptions. In Kenya and Ethiopia, programs to distribute books about puberty improved MHM knowledge and required minimal involvement from educators or parents [42–44]. Disseminating culturally responsive SRH books to schools could provide girls with standardized and comprehensive information about puberty. Government-facilitated trainings for educators could provide teachers with evidence-based, culturally appropriate MHM curricula for boys and girls. Student-led SRH school clubs could be initiated to provide peer-to-peer support for MHM.

This is one of the first studies to contextualize MHM as an environmental topic through the characterization of WASH resources and waste disposal practices. Our study also provides a snapshot into MHM experiences in Rwanda’s varied school paradigms. However, we encountered several limitations. First, this study excluded girls who dropped out of school or were absent on the day of the site visit. To gain the most inclusive sample possible, we surveyed LSS girls, since female school dropout rates increase sharply in Rwanda once girls reach SSS [20]. Second, we only collected data at 15 schools, which represents a small proportion of the 1,728 LSS schools LSS in Rwanda [21]. However, we did collect data from one school of each type (public, government aided, private) in each province to ensure that our sample represented a diversity of socioeconomic and geographical backgrounds in Rwanda. Lastly, we asked girls to report the cost of MHM on a per-cycle basis to minimize the recall period and mitigate recall bias. We then multiplied these costs by 12 to estimate the annual cost of MHM. Girls may menstruate more or fewer than 12 times per year. However, by asking about the cost of MHM per menstrual cycle, we limited the influence of recall bias that could have been introduced if we asked per annum. To minimize the impact of social desirability bias for research on the culturally sensitive topic of MHM, we explained the anonymization of survey results and the Jar Voices activity.

## Conclusion and recommendations

This study documented key challenges to MHM, including costs of menstrual products, access to WASH/sanitation facilities, and inadequate menstrual education. To bridge gaps in menstrual education, Rwanda’s national SRH curriculum could be standardized to include information about WASH and MHM. Government-facilitated workshops could train educators to teach the revised curriculum. Girls, particularly those in government-aided/public schools, could be trained on how to make re-usable pads from clean fabric to minimize the costs and risks associated with acquiring disposable sanitary products. Lastly, schools may employ low-cost interventions such as rainwater harvesting and build filtered incinerators from locally available materials to improve sanitary waste disposal and overall access to WASH/sanitary facilities. Schools could also benefit from partnerships with existing community-based organizations which are primarily focused on MHM issues in Rwanda.
